# A lightweight grape detection model in natural environments based on an enhanced YOLOv8 framework

**DOI:** 10.3389/fpls.2024.1407839

**Published:** 2024-07-25

**Authors:** Xinyu Wu, Rong Tang, Jiong Mu, Yupeng Niu, Zihan Xu, Ziao Chen

**Affiliations:** ^1^ College of Information Engineering, Sichuan Agricultural University, Ya’an, China; ^2^ Sichuan Key Laboratory of Agricultural Information Engineering, College of Information Engineering, Sichuan Agricultural University, Ya’an, China

**Keywords:** YOLOv8, grape detection, computer vision, intelligent agriculture, lightweight model

## Abstract

Grapefruit and stem detection play a crucial role in automated grape harvesting. However, the dense arrangement of fruits in vineyards and the similarity in color between grape stems and branches pose challenges, often leading to missed or false detections in most existing models. Furthermore, these models’ substantial parameters and computational demands result in slow detection speeds and difficulty deploying them on mobile devices. Therefore, we propose a lightweight TiGra-YOLOv8 model based on YOLOv8n. Initially, we integrated the Attentional Scale Fusion (ASF) module into the Neck, enhancing the network’s ability to extract grape features in dense orchards. Subsequently, we employed Adaptive Training Sample Selection (ATSS) as the label-matching strategy to improve the quality of positive samples and address the challenge of detecting grape stems with similar colors. We then utilized the Weighted Interpolation of Sequential Evidence for Intersection over Union (Wise-IoU) loss function to overcome the limitations of CIoU, which does not consider the geometric attributes of targets, thereby enhancing detection efficiency. Finally, the model’s size was reduced through channel pruning. The results indicate that the TiGra-YOLOv8 model’s mAP(0.5) increased by 3.33% compared to YOLOv8n, with a 7.49% improvement in detection speed (FPS), a 52.19% reduction in parameter count, and a 51.72% decrease in computational demand, while also reducing the model size by 45.76%. The TiGra-YOLOv8 model not only improves the detection accuracy for dense and challenging targets but also reduces model parameters and speeds up detection, offering significant benefits for grape detection.

## 1 Introduction

Grapes are heralded as the “queen of fruits” and possess considerable economic value (Chen et al., 2023). Owing to their nutritional and financial benefits, the area under grape cultivation and the scale of production have been expanding annually (Sun et al., 2022), with the global output reaching 75 million tons (Moro et al., 2021). Grape harvesting is a critical component of the grape industry’s large-scale development. Harvesting is predominantly manual, a process that requires significant labor, incurs high costs and is relatively slow (Bac et al., 2014). Estimates suggest that manually harvesting a ton of grapes costs three times more than mechanical harvesting. Hence, compared to manual picking, mechanical harvesting offers unparalleled advantages (Lin et al., 2023).

As a crucial branch of artificial intelligence, computer vision has enabled many challenging functions for traditional methods. Since their introduction, convolutional neural network models trained using deep learning methods, such as Fast-R-CNN (Girshick, 2015), Faster-R-CNN (Ren et al., 2015), YOLOv4 (Bochkovskiy et al., 2020), and SSD (Liu et al., 2016), have achieved remarkable success in computer vision. Consequently, methods based on computer vision are receiving increasing attention in technical research across various fields, particularly in remote sensing (Bai et al., 2024), transportation (Dilek and Dener, 2023), and agriculture (Tian et al., 2020). Target detection plays an essential role in harvesting robots, where the rapid and accurate identification and localization of fruits and pedicels are crucial to achieving automated harvesting (Luo et al., 2016). Although the YOLOv8 model shows improved performance over previous YOLO models in metrics like mAP, its complex structure and high computational demand render it unsuitable for deployment on endpoint devices. Therefore, achieving a lightweight structure of the YOLOv8 model without compromising its performance has become an important research topic to advance grape automated harvesting technology.

Researchers have applied traditional detection methods to detect objects in natural environments. Lin et al. (2020) proposed a novel fruit detection technique in natural settings based on a support vector machine classifier supported by color and texture features. Chaivivatrakul and Dailey (2014) introduced a plant green fruit detection technology based on texture analysis, assessing interest features in pineapples and bitter gourds. Luo et al. (2018) utilized a segmentation algorithm based on K-means clustering and practical color components to detect the cutting points of stem peduncles in overlap-ping grape clusters within unstructured vineyards. Pérez-Zavala et al. (2018) developed a method for grape berry recognition and grape bunch detection using a visible spectrum camera. However, In the face of complex natural environments, traditional target detection methods struggle to adapt to changing conditions, such as variations in lighting, occlusion and covering among detection targets, and dense distribution of objects. This situation can lead to reduced accuracy in final detection and identification, adversely affecting the recognition of grape berries, pedicels and the determination of harvesting locations.

Amid the rapid advancement and widespread adoption of deep learning, numerous methods from this domain have been applied in agricultural contexts, primarily categorized into semantic segmentation and object detection approaches (Lu and Luo, 2022). For example, within the scope of semantic segmentation, Yu et al. (2019) introduced the Mask-RCNN algorithm to generate mature fruit mask images, enhancing the efficacy of machine vision in detecting fruits for strawberry harvesting robots. Luo et al. (2015) employ improved clustering image segmentation and point-line minimum distance constraints, achieving an accuracy rate of 88.33%. Zhou et al. (2023) used an enhanced YOLACT++ model for segmenting critical structures of grapes, with the success rates of the picking point localization methods increasing by 10.95 and 81.75 percentage points. Liu et al. (2020) applied the Chan-Vese model for iterative recognition of grape clusters, attaining an average accuracy of 89.71% and a success rate of 90.91%. However, in practical applications, the achievements of target detection in natural environments based on semantic segmentation are relatively few. This arises from the extensive time needed to prepare training sets, the models’ inability to perform in real-time, and their significant size, which complicates deploying them on embedded machines in contemporary agriculture.

Applying convolutional neural networks (CNNs) for object detection for this task typically falls into two categories. The first category encompasses two-stage object detection methods: R-CNN, Fast R-CNN, and Faster R-CNN. These approaches extract object regions and then perform CNN classification and regression on those regions, constituting a detection strategy based on region proposals. For instance, Gao et al. (2020) introduced a SNAP system based on Faster R-CNN for multi-category apple detection, achieving a frame rate of 0.241s. Behera et al. (2021) presented an FR-CNN algorithm that utilizes Intersection over Union (IoU) for plant fruit prediction, reaching an accuracy of 89% in fruit yield estimation. The second category involves one-stage object detection methods, such as YOLO and SSD. Ma et al. (2024) employed an enhanced YOLOv8 model for real-time detection of apples in orchard environments, achieving an average precision of 91.4%. Wu et al. (2023) developed a stem localization method for grapes from a top-down perspective using a lightweight YOLOv5m detection algorithm based on HRNet, with a detection speed of 7.7 frames per second. It is evident that, compared to the CNN series, the YOLO series achieves comparable performance and higher computational efficiency in crop fruit detection.

Although traditional deep learning methods have improved detection accuracy, their networks are complex, necessitating substantial storage space, and are thus generally unsuitable for deployment on embedded devices. YOLOv8 is one of the fundamental models in the YOLO series, offering a new state-of-the-art model. However, due to the complexity of orchard lighting and diverse backgrounds, the YOLOv8 detection algorithm still needs to improve its object recognition. Moreover, due to the relatively large size of the YOLOv8 model, deploying it on embedded devices like harvesting robots remains challenging. To address issues of large model size, slow detection speed, and low accuracy, this paper introduces the TiGra-YOLOv8 model, a lightweight and high-precision solution designed to improve the detection efficiency of grapefruit and grape stem in the natural environment, achieving a lightweight model.

## 2 Materials and methods

### 2.1 Dataset

The grape dataset studied in this paper was collected on September 9, 2023, at the Four Seasons Vineyard in Chengdu, Sichuan Province, featuring the “Crimson” variety of grapes. We captured the images with a Canon camera, positioning the lens 0.5m to 1.5m away from the grapes. Moreover, we acquired all grape images under natural light from 12:00 to 4:00 PM. The dataset was collected in the vineyard’s natural environment, having different environmental conditions.

**Figure 1:**
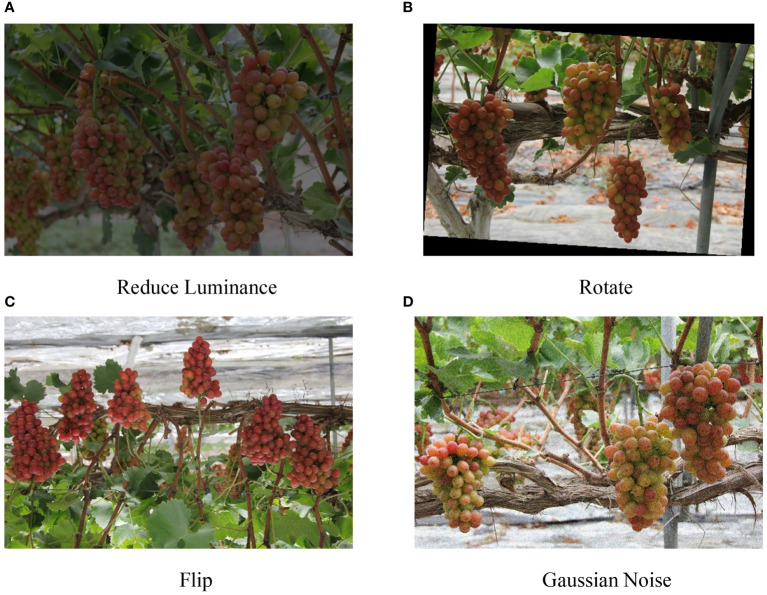
Data enhancement methods. **(A)** Reduce Luminance; **(B)** Rotate; **(C)** Flip; **(D)** Gaussian Noise.

We annotated the dataset using the professional annotation software LabelImg, marking grapefruits and grape stems with rectangular boxes (Russell et al., 2008). We labeled the fruits “grape” and the stems “grape root.” The collected dataset, after a selection process, consisted of 913 images. However, when the number of training samples is insufficient, it will often lead to overfitting of the model, which will further affect the detection effect of the model. Therefore, we expanded the original data set to 2500 through data enhancement technology, including image rotation, adding noise, flipping, and changing brightness. The data enhancement methods are shown in **Figure 1**. After image quality screening, we finally obtained 2411 data sets as training samples, which were divided according to the ratio of 7:2:1, including 1687 training sets, 482 verification sets, and 242 test sets. The original resolution of the images used for training the model was 5184x3456 pixels. To optimize the computational efficiency and ensure compatibility with the YOLOv8 model, we resized the images to 640x640 pixels. It helps standardize the input size and balance the trade-off between computational load and model performance.

### 2.2 TiGra-YOLOv8

YOLOv8 is an end-to-end convolutional neural network based on deep learning, comprising three main components: Backbone, Neck, and Head. Unlike the Anchor-based approach used in earlier models of the YOLO series, YOLOv8 introduces an Anchor-free method that achieves higher detection accuracy and speed (Lou et al., 2023). In the Backbone of YOLOv8, the model extracts image features through pooling and convolution, reducing the parameters and computational load (del Pilar Martínez-Diz et al., 2020). After upsampling and feature fusion, the Neck section sends three output results to the Head layer for loss function calculation. For the matching strategy, YOLOv8 employs the TaskAlignedAssigner method (Feng et al., 2021), which matches positive and negative samples for loss calculation based on the weighted results of classification and regression scores.

**Figure 2:**
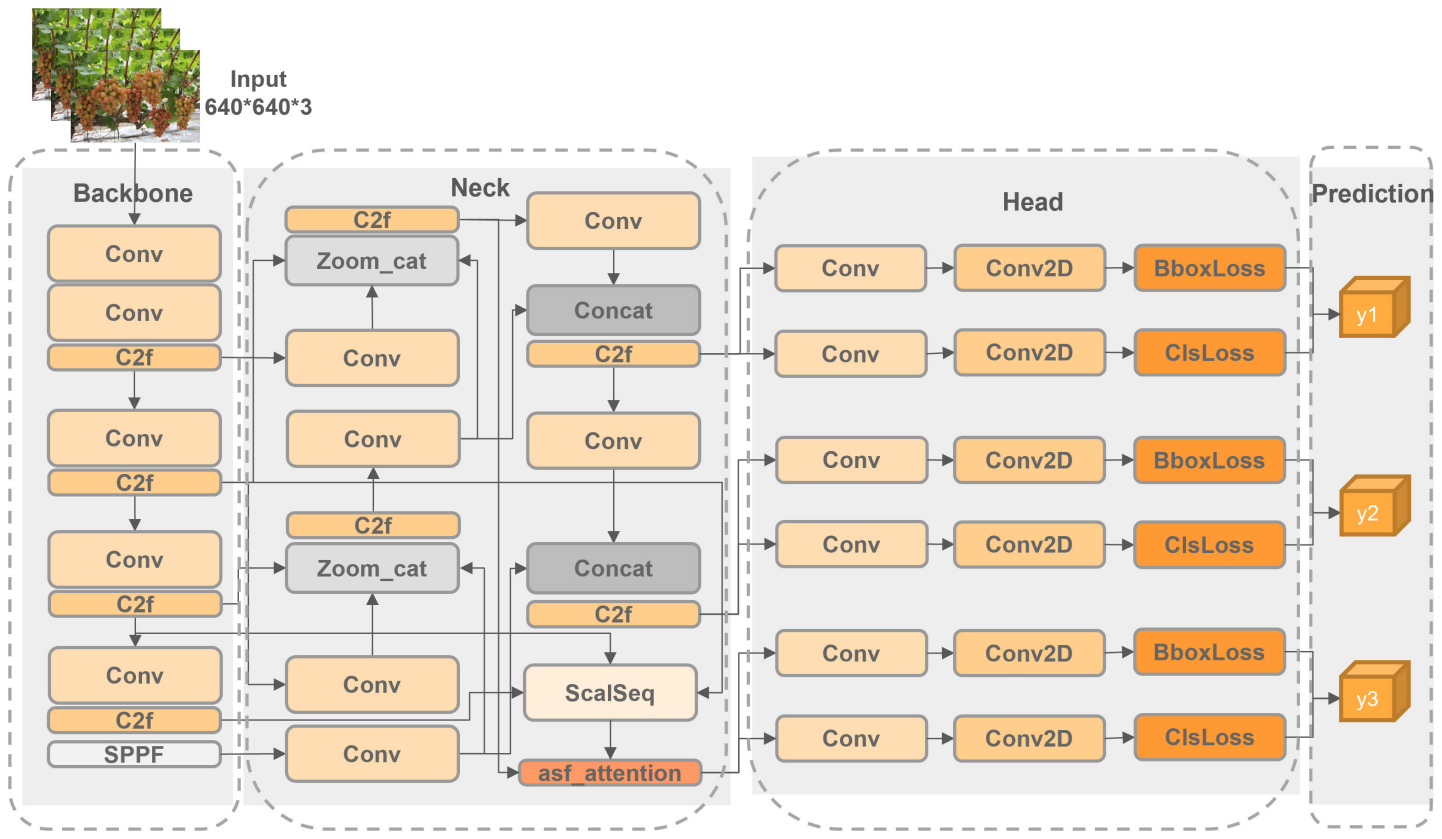
TiGra-YOLOv8 network structure.

This paper presents an enhanced TiGra-YOLOv8 model based on the YOLOv8n model. As illustrated in **Figure 2**, we have integrated the Attentional Scale Sequence Fusion module from the weighted Bi-Directional Feature Pyramid Network ASF-YOLO into the YOLO framework to improve the Neck section of YOLOv8 (Kang et al., 2023). We have adopted the Adaptive Training Sample Selection (ATSS) method for adaptive positive and negative sample selection to refine the TaskAlignedAssigner label-matching algorithm of YOLOv8 (Zhang et al., 2020). Additionally, we chose the boundary box loss based on a dynamic non-monotonic focusing mechanism (Wise-IoU) to replace the original CIoU loss function of YOLOv8 (Tong et al., 2023). Finally, by employing the Random channel pruning method, we have reduced the model’s size and parameter count while ensuring accuracy, achieving a lightweight model deployment (Liu et al., 2022).

### 2.3 Experimental detail

All models were trained under identical hardware conditions and with the same initial training parameters to ensure fairness in experimental comparisons. Mosaic data augmentation was turned off once in the last ten training cycles to better fine-tune model parameters. The specific initial training parameters are listed in **Table 1**. The model accuracy reported in this paper is based on a single training session. To ensure the reproducibility of the results, a random seed was set during the training process. Hyperparameter tuning was conducted using the validation set, and the test set’s final accuracy was evaluated.

**Table 1:** Initial training parameters.

Parameter	Form/Value
epochs	150
batch	16
close_mosaic	10
workers	8
optimizer	SGD
dropout	0.3
seed	42
lr0	0.01
lrf	0.01

The experimental environment for this study included an RTX 3090 (24G) graphics card; Python version 3.8.16; CUDA version 11.7; Torch version 1.13.1+cu117; and TorchVision version 0.14.1+cu117.

### 2.4 Evaluation Index

Mean Average Precision (mAP) is the average of the Average Precision (AP) across all categories, where AP represents the average precision for each category. The formula for calculating mAP is mAP = 1/m∑AP(i), where m is the number of categories and AP(i) is the average precision of the i-th category.


$$P=\frac{TP}{TP+FP}\times 100\%$$

$$R=\frac{TP}{TP+FN}\times 100\%$$

$$AP=\int_{0}^{1}P\left(R\right)dR\times 100\%$$

$$mAP=\frac{1}{k}\sum_{i=1}^{k}AP_{i}\times 100\%$$

mAP(0.5) and mAP(0.75) refer to the average precision of each category at Intersection over Union (IoU) thresholds more significant than 0.5 and 0.75, respectively. mAP(0.5:0.95) denotes the mAP across different IoU thresholds, ranging from 0.5 to 0.95, with a step size 0.05. Loc denotes localization errors, Dupe indicates duplicate detection errors, and FalsePos represents overall error metrics, including duplicate detection, incorrect classification, localization errors, and background confusion. Model Size, Parameters, and GFLOPS, respectively, refer to the storage size, number of parameters, and computational cost of the model, commonly used to evaluate the size of a network model. FPS, or frames per second, measures the number of images that can be detected per second and is used to assess the detection speed of the network model.

## 3 Model Improvement

### 3.1 Neck

#### 3.1.1 ASF network structure

**Figure 3:**
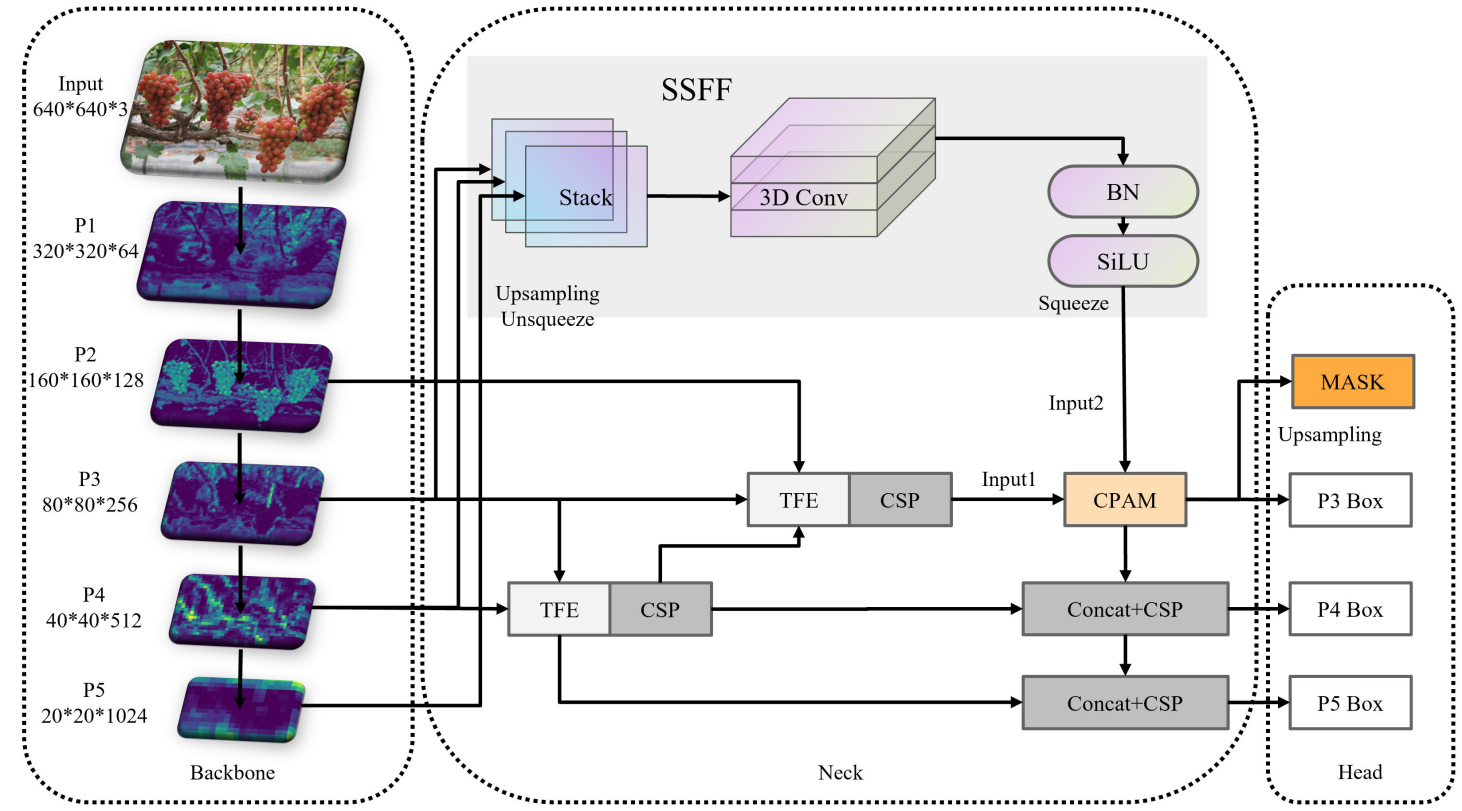
ASF module structure.

Integrating the Attentional Scale Sequence Fusion (ASF) module from ASF-YOLO into the YOLO framework to enhance the Neck component of YOLOv8 enables a more effective combination of high-dimensional information from deep feature maps with detailed information from shallow feature maps. The ASF feature extraction network consists of Scale Sequence Feature Fusion (SSFF), Triple Feature Coding (TPM), and Channel and Position Attention Model (CPAM) modules. Its network structure is depicted in **Figure 3**.

During forward propagation, the SSFF module merges feature maps from different spatial scales, preserving scale-invariant features and enhancing the representation of multi-scale information. This process ensures that the model can effectively detect objects of varying sizes and shapes. The TFE module further processes these feature maps by adjusting their scales and combining them into a unified feature map. This helps in accurately identifying small and overlapping targets by providing a comprehensive multi-scale context. The CPAM module applies attention mechanisms to focus on relevant spatial and channel-wise information, enhancing the localization and classification of small targets like grape stems. This selective attention improves the model’s ability to differentiate between similar objects and detect fine details.

During backward propagation, the enhanced feature representation and selective attention mechanisms provided by ASF-YOLO result in more stable and informative gradients during backward propagation. This leads to more efficient parameter updates and faster convergence, Stabilizing the gradient flow. The multi-scale fusion and attention mechanisms ensure that the model learns robust and discriminative features, which are essential for accurate detection. The gradients calculated for these features are more informative, leading to better weight adjustments and improved model performance.

#### 3.1.2 SSFF module

The SSFF module effectively merges feature maps from different spatial scales (e.g., layers P3, P4, P5), capturing scale-invariant features that remain consistent despite variations in size or shape. This capability is crucial for detecting grapes of various sizes within a cluster, as it enables the model to recognize both small individual grapes and larger grape clusters.

The grape-scaled images inputted into the SSFF can be obtained by the following method:


$$F_{0}\left(w,h\right)=G_{0}\left(w,h\right)\times f\left(w,h\right)$$

$$G_{\sigma }\left(w,h\right)=\frac{1}{2\pi \sigma^{2}}{\text{e}}^{-\left(\omega^{2}+h^{2}\right)/2\sigma^{2}}$$

where f(w,h) denotes a 2D input image with width *w* and height *h*. $F_{0}\left(w,h\right)$ is generated by smoothing under a 2D Gaussian filter through convolutions. *σ* represents the standard deviation parameter of the 2D Gaussian filter used for convolution.

#### 3.1.3 TFE module

**Figure 4:**
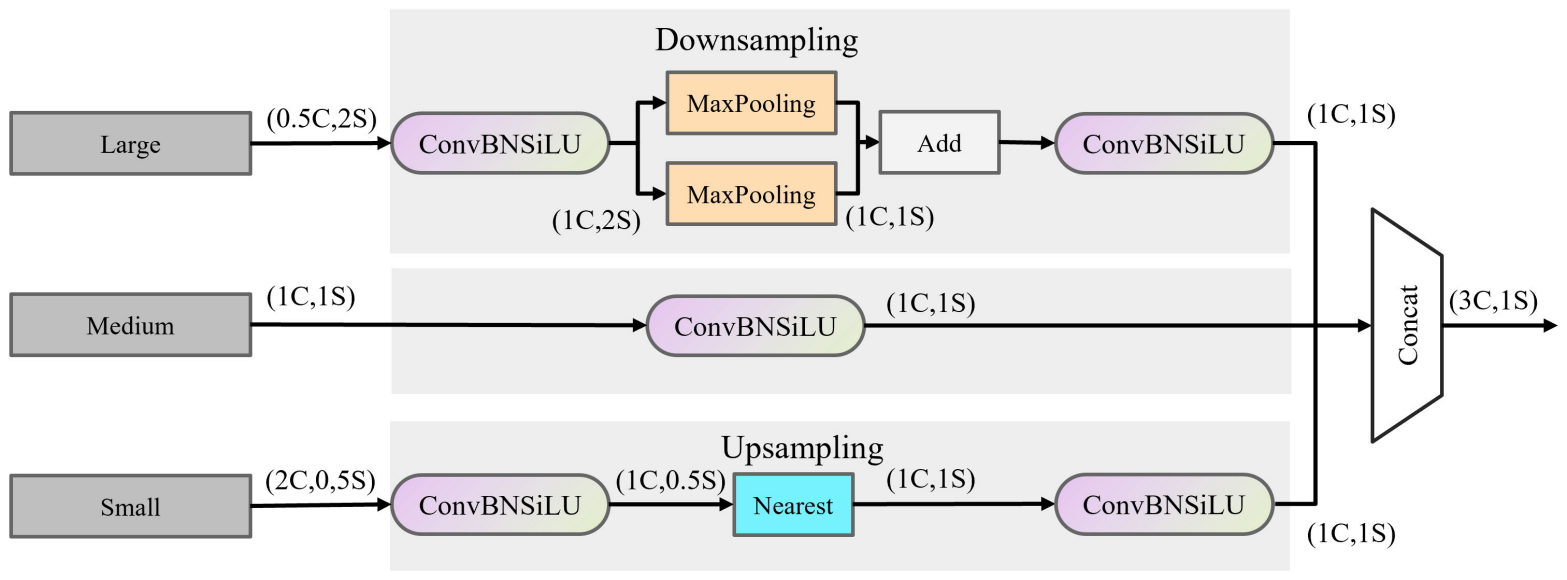
TFE module structure.


The TFE module enhances the detection of small, densely overlapping objects like individual grapes within a cluster by examining and comparing image variations across different scales. By magnifying and combining feature maps from large, medium, and minor scales, the TFE module ensures that even the smallest details are captured and emphasized. The module adjusts the scales of input feature maps (large, medium, and small) to a standard scale, allowing for effective fusion and comparison. This adaptation aids the model in recognizing grapes irrespective of their size, leading to improved detection accuracy for both small and large grape clusters. The TFE module is illustrated in **Figure 4**.

$$F_{TFE}=Concat\left(F_{l},F_{m},F_{s}\right)$$

$F_{TFE}$ represents the output feature map of the TFE module, with $\;F_{l},F_{m},F_{s}\;$ enoting the large, medium, and small-sized feature maps, respectively. The information output by the TFE is integrated into each feature map branch through the PANet structure, combined with the multi-scale information output by the SSFF module into the P3 branch for subsequent attention network feature extraction.

#### 3.1.4 CPAM module

**Figure 5:**
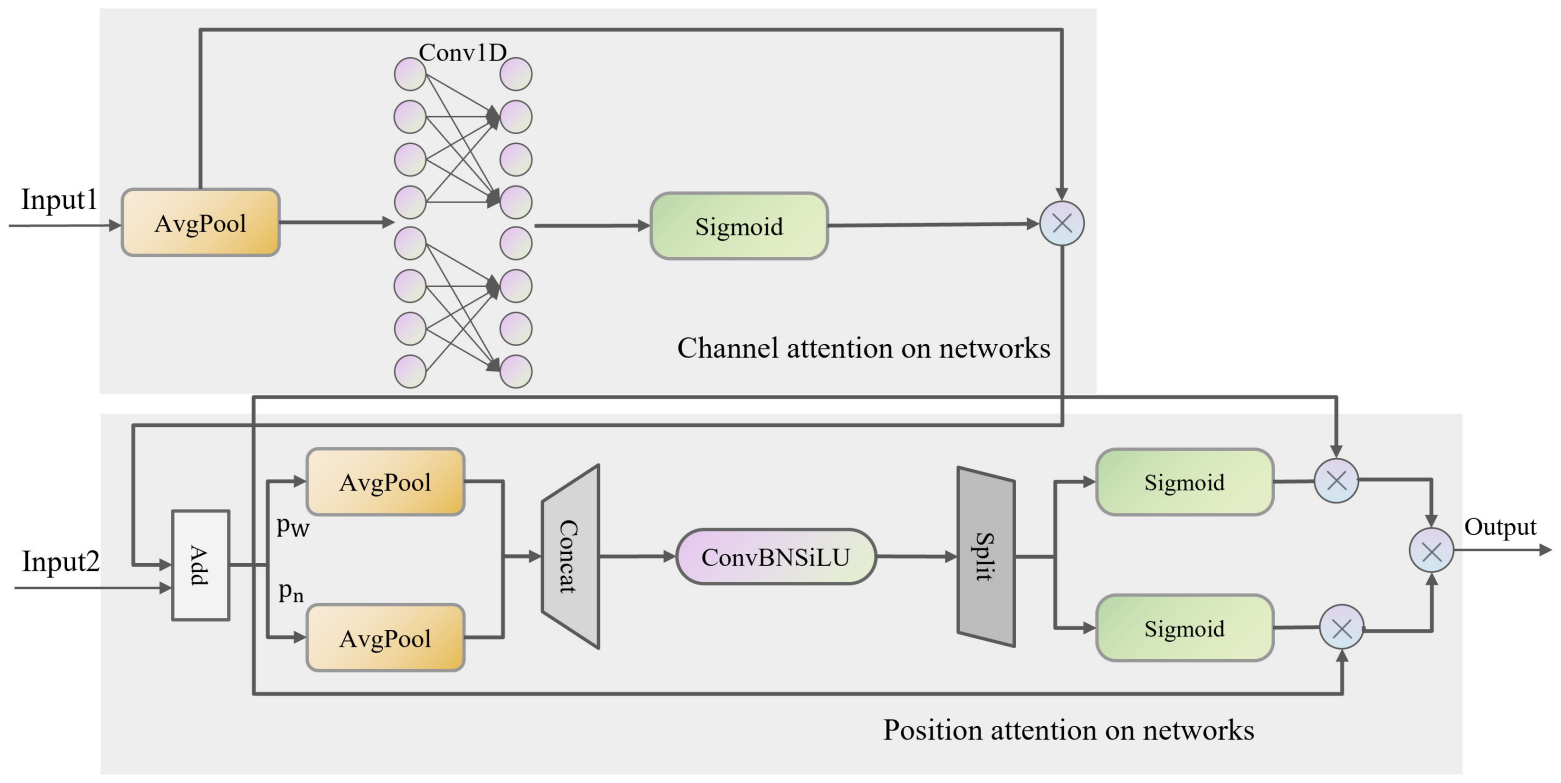
CPAM module structure.

The CPAM module employs channel attention mechanisms to focus on the most relevant channels in the feature maps. By prioritizing channels containing critical information about grape clusters and fruits, the model can more accurately identify and classify these objects. This is particularly useful for distinguishing closely packed grapes from background elements. The CPAM module also applies spatial attention mechanisms to highlight significant spatial regions within the feature maps. This enhances the model’s ability to localize small targets like individual grapes and grape stems, improving detection precision and reducing false positives. By integrating inputs from the TFE and SSFF modules, the CPAM module ensures that both high-order multi-scale features and detailed spatial information are utilized. This comprehensive integration enables the model to effectively detect and differentiate grape clusters and individual grapes in complex and cluttered scenes. The structure of CPAM is shown in **Figure 5**.

### 3.2 Label-matching policy

**Figure 6:**
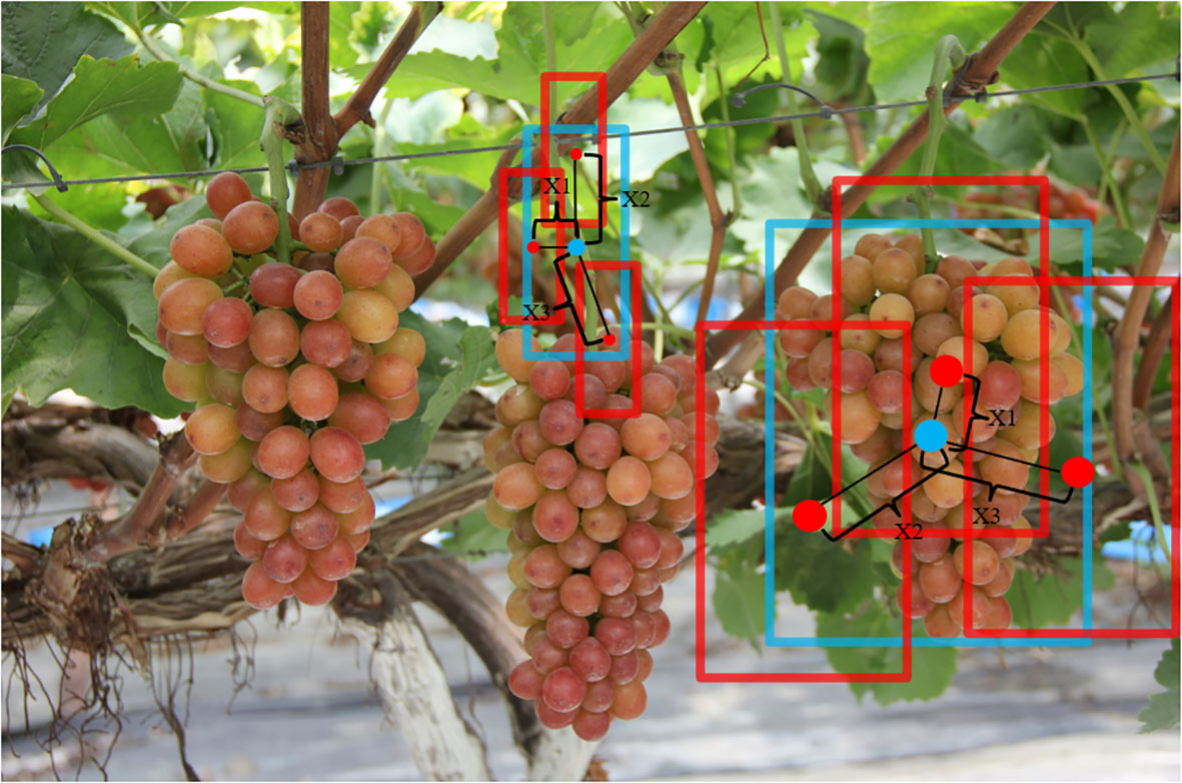
Grape stem and fruit pre-selection frame.

YOLOv8 employs the TaskAlignedAssigner label-matching strategy, which selects positive samples based on a weighted score of classification and regression scores. The TiGra-YOLOv8 model this paper introduces utilizes the Adaptive Training Sample Selection (ATSS) label-matching algorithm. As illustrated in **Figure 6**, blue boxes represent the ground-truth boxes (gt), and red boxes represent the anchor boxes (anchor). ATSS calculates the distances x1, x2, x3 between each anchor and its center point if a feature map generates three anchors for grape stems and fruits. If x1< x2< x3, it selects the anchors corresponding to the shorter distances x1 and x2 as candidate positive samples. For L levels of feature maps, L*2 candidate positive samples are chosen. Subsequently, the IoU between each candidate positive sample and the gt is calculated, followed by the calculation of the mean and variance of the IoUs. The threshold for selecting positive samples is t = m + g, where m is the mean and g is the variance. Finally, based on the threshold t for each layer, the actual positive samples to be included in the training are selected from the candidate positive samples.

During forward propagation, ATSS optimizes the selection of positive and negative samples, ensuring that the model learns features from more representative samples. These features are more indicative of the actual objects, improving the model’s ability to detect and distinguish grape clusters and individual grapes. By focusing on adaptively chosen samples, the model can better localize and classify objects, leading to higher detection accuracy and fewer false positives. This is crucial for identifying small, densely packed objects such as grapefruits within clusters. In the backward propagation process, the improved selection of training samples results in more stable and informative gradients, enhancing the efficiency of parameter updates and speeding up model convergence. By ensuring a more balanced distribution of positive and negative samples across different object scales, ATSS facilitates more effective learning of multi-scale features, which is particularly beneficial for accurately detecting both small individual grapes and larger grape clusters.

### 3.3 IoU loss

In the YOLOv8 network, CIoU is the bounding box regression loss function. It calculates the loss by considering the overlap area between predicted boxes and ground-truth boxes, the distance between their center points, and the aspect ratio, with the formula detailed below:


$${ℒ}_{CIoU}=1-IoU+\frac{\rho^{2}\left(b,b^{gt}\right)}{c^{2}}+a\nu$$

CIoU employs a monotonic focusing mechanism aimed at enhancing the fitting capability of the bounding box loss. However, when the object detection training set contains low-quality examples, indiscriminately reinforcing the regression for low-quality examples could hinder the improvement of model detection performance (Zheng et al., 2020). Consequently, this paper adopts a dynamic non-monotonic focusing mechanism loss function, WIoU, which constructs distance attention based on distance metrics, resulting in WIoUv1. The formula for this loss function is as follows:


$${ℒ}_{WIoUv1}\equiv R_{WIoU}{ℒ}_{IoU}$$

$$R_{WIoU}=\exp\left(\frac{{\left(x-x_{gt}\right)}^{2}+{\left(y-y_{gt}\right)}^{2}}{{\left(w_{g}^{2}+H_{g}^{2}\right)}^{*}}\right)$$

WIoUv3 defines an outlier degree to describe the quality of anchor boxes, assigning smaller gradient gains to anchors with a more considerable outlier degree. This effectively prevents harmful gradients from low-quality examples, allowing the loss function to focus more on samples of average quality and improve the overall model performance.


$$\beta =\frac{{ℒ}_{IoU}^{*}}{{ℒ}_{IoU}}\in \left[0,+\infty \right]$$

$${ℒ}_{WIoUv3}=\gamma {ℒ}_{WIoUv1}$$

$$\gamma =\frac{\beta }{\delta a^{\beta }}$$

During training, WiseIoU enhances the model’s ability to learn from complex and varied object shapes by providing more informative feedback on the quality of predictions. This leads to better localization and classification performance, as the model is trained to optimize for a metric that closely aligns with the practical requirements of accurate object detection in vineyards. The improved feedback mechanism helps the model to fine-tune its predictions more effectively, reducing the number of false positives and false negatives.

In the evaluation phase, WiseIoU offers a more reliable and discriminative measure of the model’s detection performance. Traditional IoU can sometimes overestimate the accuracy of detection when dealing with minor or partially overlapping objects. WiseIoU mitigates this issue by providing a more detailed and context-aware assessment, ensuring that the model’s performance metrics more accurately reflect its real-world detection capabilities. Moreover, WiseIoU contributes to the robustness of the detection model by improving its generalization across different environmental conditions and image qualities. By accounting for various object shapes and contextual nuances, the model trained with WiseIoU is better equipped to handle variations in lighting, occlusion, and background clutter commonly encountered in vineyard images. This leads to more consistent and reliable detection results, enhancing the overall effectiveness of the model in practical agricultural settings.

### 3.4 Channel pruning

This study employs the Random channel pruning(RCP) method, offering a novel approach to estimating the contribution of neurons (filters) to the final loss. During training, the contribution to the final loss is analyzed based on average gradients and weight values, and iteratively, neurons with lower scores are removed. The model takes a trained network as input during the pruning process and prunes it at a reduced learning rate during iterative fine-tuning. Following fine-tuning, the model’s accuracy is restored.

The RCP algorithm contributes to the robustness and adaptability of the detection model, encouraging the model to learn redundant and complementary features across different channels. This redundancy ensures that the model can still perform accurately even if specific channels are removed. For the detection of grape clusters and fruits, this means that the model can maintain high detection accuracy despite variations in grape size, shape, occlusion, and lighting conditions, which are commonly encountered in vineyard images. RCP also aids in mitigating overfitting. Reducing the number of channels and, thus, the model’s capacity helps prevent the model from memorizing the training data. Instead, it promotes the learning of generalizable features that are applicable to unseen test data. This characteristic is particularly beneficial for detecting grape clusters and fruits, where the model must generalize well across different vineyard locations and conditions. By focusing on essential features and discarding redundant ones, RCP ensures that the model remains effective and accurate in detecting grape clusters and fruits in a wide range of scenarios.

## 4 Results

### 4.1 Comparison of neck

In the Neck section, this paper implements six distinct enhancements to the YOLOv8n model: YOLOv8n-CARAFE, YOLOv8n-EfficientRepBipan, YOLOv8n-GDFPN, YOLOv8n-GoldYolo, YOLOv8n-HSPAN, and YOLOv8n-ASF. **Table 2** displays the comparative results of these network models in terms of mAP, FalsePos, and ModulSize.

**Table 2:** Neck end network model improvement comparison.

Model	mAP(0.5)	mAP(0.5:0.95)	FalsePos	ModulSize (M)
YOLOv8n(Baseline)	0.900	0.557	6.89	5.9
YOLOV8n-CARAFE	0.909	0.593	5.83	6.2
YOLOv8n- EfficientRepBipan	0.915	0.569	5.16	5.6
YOLOv8n-GDFPN	0.914	0.576	5.83	6.5
YOLOv8n-GoldYolo	0.910	0.580	5.38	11.8
YOLOv8n-HSPAN	0.910	0.583	5.99	4.2
**YOLOV8n-ASF**	**0.916**	**0.590**	**5.01**	**6.0**

The bold values in the table are the best methods in the experiment and their corresponding experimental data.

Data analysis from **Table 2** reveals that YOLOv8n-ASF achieves an mAP(0.5) and mAP(0.5:0.95) of 1.78% and 5.92% higher than the Baseline, respectively. Simultaneously, FalsePos and ModulSize are reduced by 27.28% and 1.69% compared to the Baseline. Although YOLOv8n-EfficientRepBipan and YOLOv8n-GDFPN also show a significant improvement in mAP compared to the Baseline, its FalsePos and ModulSize are higher, indicating that while YOLOv8n-GoldYolo may increase accuracy in grape detection, it results in more detection errors and has a larger storage volume, making it less suitable for deployment in embedded devices like automated grape harvesting robots. Among the improved network models, YOLOv8n-ASF demonstrates the best comprehensive improvement in grape detection.

### 4.2 IoU loss function comparison experiment

To investigate the impact of loss functions on the performance of the improved model, we set YOLOv8n as the Baseline and a model with enhancements to the Neck section and label matching strategy, YOLOv8n+ASF+ATSS, as Model A. This paper compares the mAP values and FalsePos of five commonly used IoU loss functions: IoU, EIoU, MDPIoU, and Wise-IoU. The comparative results of these loss functions are shown in the table.

**Table 3** shows that the YOLOv8n model using the Wise-IoU loss function achieves higher detection accuracy. Compared to the original YOLOv8n model using the CIoU loss function, mAP(0.5) and mAP(0.5:0.95) increased by 3.11% and 7.36%, respectively, with FalsePos decreasing by 35.84%. Relative to Model A, which improved both the Neck section and label matching strategy, mAP(0.5) and mAP(0.5:0.95) increased by 1.19% and 1.35%, respectively, with FalsePos decreasing by 18.89%. These results indicate that using the Wise-IoU loss function can stabilize the model’s bounding box regression and improve prediction accuracy.

**Table 3:** Comparison of model performance after improved loss function.

Model	mAP(0.5)	mAP(0.5:0.95)	FalsePos
YOLOv8n(Baseline)	0.900	0.557	6.89
YOLOv8n+ASF+ATSS(A)	0.917	0.590	5.45
A+IoU	0.919	0.589	5.13
A+EIoU	0.926	0.602	4.96
A+MPDIoU	0.926	0.592	4.30
A+ShapeIoU	0.922	0.588	5.32
**A+Wise-IoU**	**0.928**	**0.598**	**4.42**

The bold values in the table are the best methods in the experiment and their corresponding experimental data.

### 4.3 Ablation experiment

Ablation experiments were conducted to validate the effectiveness of the Attentional Scale Sequence Fusion (ASF) module, Adaptive Training Sample Selection (ATSS) label matching strategy, and the enhanced Wise-IoU loss function within the TiGra-YOLO network model for grape detection. The results, showcasing mAP values across various IoU thresholds and comparative metrics for different error types, are presented in **Table 4**, where “×” denotes the absence of the corresponding enhancement module and its presence otherwise.

**Table 4:** Comparison of ablation experimental indexes.

ASF-YOLO	ATSS	Wise-IoU	mAP(0.5)	mAP(0.75)	mAP(0.5:0.95)	Loc	Dupe	FalsePos
×	×	×	0.900	0.547	0.557	5.69	0.75	6.89
√	×	×	0.916	0.588	0.590	4.52	0.62	5.01
√	√	×	0.917	0.605	0.590	4.89	0.46	5.45
**√**	**√**	**√**	**0.928**	**0.619**	**0.598**	**4.57**	**0.28**	**4.42**

× means that this module was not used in this round of experiment, √ means that this module was used in this round of experiment.

The bold values in the table are the best methods in the experiment and their corresponding experimental data.

In **Table 4**, the mAP values for TiGra-YOLOv8 across different IoU ranges were 0.928, 0.619, and 0.598, respectively. These values represent an increase of 3.11%, 13.16%, and 7.36% over the original YOLOv8n (Baseline). Moreover, localization errors (Loc), duplicate detection errors (Dupe), and overall error metrics (FalsePos) decreased by 19.68%, 62.66%, and 35.84% compared to the original YOLOv8n model. This indicates that the TiGra-YOLOv8 model significantly enhances the accuracy of grapefruit and stem detection. This improvement can be attributed to the ASF module, which increases detection precision for small targets like grape stems by filtering out irrelevant background noise and retaining valuable information for object detection.

Furthermore, the ATSS matching strategy effectively filters positive and negative samples. It is an adaptive selection method that divides training samples based on the statistical features (variance and mean) of grapes and their stems. Finally, the Wise-IoU, employing a dynamic non-monotonic focusing mechanism for bounding box loss, optimizes model performance based on the overlap between predicted and actual grape stems, assessing accuracy and providing a gradient gain allocation strategy focused on anchors of average quality to enhance detector performance.

### 4.4 Channel pruning experiment

This study conducted experiments on six commonly used pruning methods, setting YOLOv8n as Baseline1 and YOLOv8n+ASF+ATSS+WiseIoU as Baseline2. The pruning rate (speed_up) was set to 2.0, with global_pruning set to False, meaning that the number of pruned channels per layer was roughly consistent. Performance metrics such as model size, parameter count, computational cost, FPS, and mAP were compared, as shown in **Table 5**.

**Table 5:** Comparison of indicators after pruning.

Prune Method(2.0x)	ModulSize (M)	Parameter	GFLOPS	FPS	mAP(0.5)
YOLOv8n(Baseline1)	5.9	3.20M	8.7	1066.3	0.900
(Baseline2)	6.0	3.05M	8.5	863.6	0.928
L1	3.2	1.53M	4.2	1144.1	0.928
Lamp				1144.4	0.915
Group_taylor				1144.9	0.922
Group_norm				1142.3	0.920
Group_hessian				1140.8	0.925
**Random**				**1146.2**	**0.930**

The bold values in the table are the best methods in the experiment and their corresponding experimental data.

Comparing the six pruning models with the Baselines revealed that Random achieved the best overall performance metrics. Compared to Baseline1, it improved mAP by 3.33% and FPS by 7.49%. Compared to Baseline2, mAP increased by 0.21% and FPS by 32.72%, while model size, parameter count, and computational cost were reduced by 50% compared to both Baseline models.

To explore the impact of the pruning rate on model performance, the study chose the Random pruning method and experimented with pruning rates of 1.5, 1.7, and 2.0. A pruning rate of 1.5 implies that the computational cost of the pruned model is 2/3 of the original model, meaning that 1/3 of the original channel connections were removed from the network. Similarly, pruning rates of 1.7 and 2.0 indicate that the computational cost of the pruned model is 1/1.7 and 1/2 of the original, respectively. After pruning, the network underwent fine-tuning to compensate for lost connections, restoring accuracy and improving overall network performance. The comparative results are presented in **Table 6**.

**Table 6:** Comparison of different pruning rate indicators.

speed_up	ModulSize (M)	Parameters	GFLOPS	FPS	mAP(0.5)
YOLOv8n(Baseline1)	5.9	3.20M	8.7	1066.3	0.900
Baseline2	6.0	3.05M	8.5	863.6	0.928
1.5x	4.1	2.02M	5.6	1028.4	0.929
1.7x	3.6	1.79M	5.0	1063.5	0.931
**2.0x**	**3.2**	**1.53M**	**4.2**	**1146.2**	**0.930**

The bold values in the table are the best methods in the experiment and their corresponding experimental data.

Analysis of **Table 6** shows that the Random pruning method achieved the highest mAP and FPS at a pruning rate of 2.0, with the smallest model size, parameter count, and computational cost. The value of mAP(0.5) increased compared to the pre-pruning model and both Baselines, indicating that this pruning method optimally enhances the comprehensive performance of the model at a pruning rate of 2.0.

### 4.5 Network model comparison experiment

**Figure 7:**
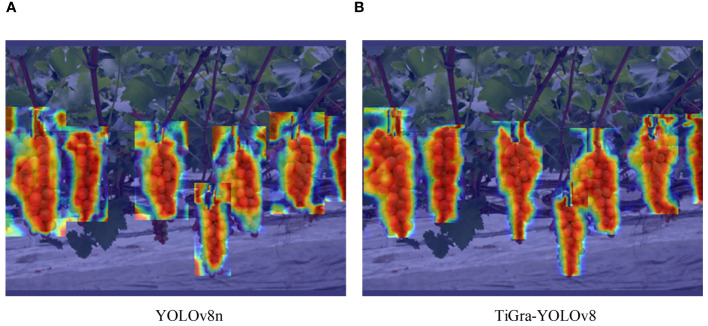
Comparison of detection heat maps. **(A)** YOLOv8; **(B)** TiGra-YOLOv8.

This experiment contrasts the detection heatmaps of YOLOv8 and TiGra-YOLOv8, as shown in **Figure 7**.

From the heat maps of A and B in **Figure 7**, it can be seen that compared to the original YOLOv8 model’s heatmap, the improved model’s heatmap exhibits more distinct edges and contours, with a more concentrated focus on the centers of the target objects.This indicates that the TiGra-YOLOv8 model proposed in this paper can better focus on the features of detection targets, thereby enhancing detection accuracy.

We also compared the improved model with existing models, such as YOLOv5n, YOLOv7-tiny, YOLOv8n, and YOLOv9-c, with the results presented in **Table 7**.

**Table 7:** Comparison of indicators of different models.

Model	mAP(0.5)	Parameters	GFLOPS
YOLOv5n	0.904	1.90M	4.5
YOLOv7-tiny	0.895	12.3M	13.1
YOLOv8n	0.900	3.20M	8.7
YOLOv9-c	0.892	25.30M	102.1
**TiGra-YOLOv8(ours)**	**0.930**	**1.53M**	**4.2**

The bold values in the table are the best methods in the experiment and their corresponding experimental data.

According to the table, compared to YOLOv5n, the mAP(0.5) increased by 2.87%, while Parameters and GFLOPS decreased by 19.47% and 6.67%, respectively. Compared to YOLOv7-tiny, the mAP(0.5) increased by 3.91%, with Parameters and GFLOPS decreasing by 87.56% and 67.94%, respectively. Compared to YOLOv8n (Baseline), the mAP(0.5) increased by 3.33%, with Parameters and GFLOPS decreasing by 52.19% and 51.72%, respectively. Compared to the currently available YOLOv9-c model, the mAP(0.5) increased by 4.26%, with Parameters and GFLOPS decreasing by 93.95% and 95.88%, respectively.

The detection accuracy, parameter count, and computational load for grape recognition in natural environments have improved compared to both previous and the most recently proposed YOLO series models.

## 5 Discussion

This study proposes a lightweight detection method based on YOLOv8. It attains a detection accuracy of 93% while reducing the model size by half. Specifically, the integration of ASF-YOLO into the neck of the TiGra-YOLOv8 model plays a crucial role in enhancing feature aggregation and improving the model’s capability to capture intricate details within images, thereby contributing significantly to the overall improvement in precision. The adoption of the ATSS matching strategy provides a dynamic approach to selecting positive and negative samples during the training process. It ensures that the model learns from a representative set of training samples, leading to more robust learning and better generalization. The utilization of the Wise-IoU loss function considers both the overlap and the distance between predicted and ground truth boxes. This dual consideration ensures that the model predicts the location of objects with greater accuracy. The implementation of the Random pruning algorithm is instrumental in reducing the model’s size by eliminating redundant and less significant parameters while preserving critical features. In fact, the reduced complexity can lead to more focused learning, as evidenced by the 4.1.4 observed increase in precision post-pruning.

The significant improvements in detection accuracy and reduction in model size achieved by the TiGra-YOLOv8 model have important implications for its potential application in agricultural settings. The enhanced precision of the model, which allows for more accurate identification of grapefruits and stems, can improve the efficiency of grape harvesting processes and reduce waste. The observed reduction in model size, achieved through the implementation of the Random pruning algorithm, indicates a promising direction towards making the model more computationally efficient. This reduction in complexity suggests the potential for the model to be adapted for deployment on devices with limited computational resources. However, additional work is required to evaluate the performance and feasibility of the TiGra-YOLOv8 model on such low-resource hardware.

The results of this study demonstrate the advantages of our approach when compared to those reported in the existing literature. Traditional machine learning algorithms, commonly used in earlier studies, need help to adapt to varying environmental conditions and typically exhibit lower accuracy. Additionally, instance segmentation models, while precise, are often large and computationally intensive, posing challenges for deployment on resource-constrained devices. The prediction accuracy of the TiGra-YOLOv8 model on this dataset is better than that of classical object detection models such as YOLOv5 and YOLOv8, which indicates that the model has particular potential in dealing with such tasks.

## 6 Conclusion and prospect

In this study, aimed at recognizing grapefruits and their stems in natural environments, we constructed a dataset from self-captured images of grapefruits and stems, considering the complex backgrounds, density, and occlusions typical of orchard settings. We designed a lightweight object detection model, TiGra-YOLOv8, incorporating the ASF module into the YOLOv8 network structure. The model also features modifications to the IoU loss function and the positive and negative sample matching strategy, enhancing detection accuracy. Furthermore, model size was reduced through channel pruning. This lightweight approach is significant for deploying the model on mobile devices. In summary, the TiGra-YOLOv8 model achieved a detection accuracy of 93%, with a model size of 3.2M, a parameter count of 1.53M, a computational cost of 4.2 GFLOPS, and an FPS of 1146.2. Compared to YOLOv5 and other models, TiGra-YOLOv8 boasts higher detection accuracy and lower model parameters.

Despite the positive findings of this study, there are certain limitations. Firstly, it should be noted that the dataset used in this study was derived solely from a single crop species, thus necessitating future testing and validation on more diverse datasets. Secondly, when deploying and applying the model in practice, practical factors such as device compatibility and real-time performance need to be taken into consideration. Additionally, although model pruning techniques have successfully reduced model complexity, further optimization is still necessary to accommodate a broader range of application scenarios. In future research, we will explore mobile deployment of the model and deploy the TiGra-YOLOv8 model on small computing devices. Additionally, we plan to collect more diverse grape datasets and train a more generalized TiGra-YOLOv8 model.

In summary, the lightweight detection method based on YOLOv8 proposed in this study demonstrated exceptional performance in detecting grape fruit and grape stalk, thereby offering a novel technical approach for agricultural automation and intelligence. Future studies will further investigate the model’s generalization ability and practical application potential.

## Data availability statement

The raw data supporting the conclusions of this article will be made available by the authors, without undue reservation.

## Author contributions

XW: Conceptualization, Data curation, Formal analysis, Methodology, Software, Visualization, Writing – original draft, Writing – review & editing. RT: Conceptualization, Data curation, Formal analysis, Software, Validation, Visualization, Writing – review & editing. JM: Project administration, Writing – review & editing. YN: Investigation, Writing – review & editing. ZX: Writing – review & editing. ZC: Validation, Writing – review & editing.
